# Effect of performance based financing home visiting on the use of modern methods of contraception in the kumbo east health district, Cameroon

**DOI:** 10.1186/s40834-016-0030-5

**Published:** 2016-08-31

**Authors:** Thomas Obinchemti Egbe, Julius Atashili, Emmanuella Talla, Mary Bih Suh Atanga

**Affiliations:** 1 Department of Obstetrics and Gynecology, Faculty of Health Sciences, University of Buea, P.O. Box 63, Buea, Cameroon; 2 Department of Public Health, Faculty of Health Sciences, University of Buea, P.O.Box 63, Buea, Cameroon; 3 Delegation of Public Health, North West Region, Cameroon; 4 Department of Nursing and Midwifery, Faculty of Health Sciences, University of Bamenda, Bambili, Cameroon

**Keywords:** Performance based financing, Utilization, Modern methods of contraception, Women of childbearing age, Medroxyprogesterone acetate

## Abstract

**Background:**

The use of modern methods of contraception (MMC) as defined by the World Health Organization still remains a challenge in most of Sub-Saharan Africa. Performance Based Financing (PBF) home visit was introduced in 2012 to increase the use of those modern methods in the Kumbo East Health District (KEHD), Cameroon.

We determined the utilization rates of MMC in areas in the KEHD with PBF home visits compared with those areas with no home visits.

**Methods:**

This was a cross sectional study carried out in the KEHD during the period February 1 to May 31, 2015. A multistage cluster sampling method was used to recruit 262 and 221 women aged 15–49 years in the intervention and nonintervention health areas, respectively. A structured, closed ended questionnaire was used.

**Results:**

The average age of women was similar in both groups 30.40 (SD 8.57); median 30 years in the intervention group and 30.49 (SD 7.84); median 30 years in controls. Most participants in the intervention health area (60.3 %) used modern methods of contraception compared to 46.6 % of those in the control group (aOR: 1.75 %; 95 % CI: 1.212.53) and the most commonly used MMC was medroxyprogesterone in the intervention group and condoms in the control area.

**Conclusion:**

Utilization of MMC was significantly higher in the area with PBF home visits than in areas without that service. While this indicates that the intervention has benefit, there may be other contributing factors.

## Background

Over half of the 278,000 maternal deaths in 2010 occurred in Sub-Saharan Africa [1, 2]. Rates of unintended pregnancy in Cameroon in 2013 totaled 490,000 and was attributable in large part to low utilization of contraception, particularly modern methods [3–6]. These latter include combined hormonal contraceptives, progestin-only pills, contraceptive injections, intrauterine devices, implants, male and female condoms and permanent contraception for both men and women. On the other hand, non-modern methods (behavior methods) include fertility awareness and coitus interruptus [7]. Progress has been made in the use of contraception in Sub Saharan Africa overall and in Cameroon in particular. However, in 2011 only 23.4 % of the population of Cameroon used any contraception and 14.1 % used MMC [5].

In order to improve utilization of contraception, a performance based financing [PBF] pilot project was introduced in the Kumbo East Health District (KEHD) in 2012. The KEHD has a surface area of about 1,087 km^2^, 20 health areas and a population of 179 789 inhabitants of which 43 689 are women of child bearing age. The KEHD has a hilly terrain with towns located on hills and valleys and there are fast flowing streams which render the district inaccessible all year round. Local community radios such as the Bui Community radio, Helenkris radio, City Community radio and radio Evangelium broadcast in the district, but many women in the study groups are farmers and do not have time to listen to radio-broadcast public health messages.

For this project, contracts were signed and nurses were trained to provide women education in their homes about contraception and prevention of sexually transmitted infections. They were taught about the reproductive cycle, mechanism of action for each of the methods. The training was designed to enable them to tell women about each method, including its effectiveness, medical eligibility, cost side effects and safety while appreciating the woman’s preference, fertility desires and risks of STDs [8, 9].

The women then returned to their homes, in the intervention areas, in the District and went from house to house to educate women about their STD risks and their options and to refer them to the nearest hospital for counseling and method initiation [9].

In the Kumbo East health district, condoms are free and available at the pro-pharmacies in Government health facilities while the other methods are also subsidized at the Government health facilities. The average cost of contraceptives was 2.63 USD; range 0.17–8.62 USD. The majority of participants used male condoms and the injectable that cost 0.17 USD, respectively. The monthly income of the population ranged between 21.83 and 94.54 USD [10].

The aim of this study was to estimate the effect of home visits on the use of modern methods of contraception by women of childbearing age in the Kumbo East Health District, Cameroon by comparing the rates of use in KEHD in areas served by home visits compared to those areas without home visits. The distances between health facilities in both study groups were similar.

## Methods

This cross sectional comparative study was approved by Faculty of Health Science Institute Review Board of the University of Buea, Cameroon and the Regional Delegation of Public Health of the North West. Women aged 15–20 years of age signed assent forms and agreement was obtained from their guardian/parent; women age 21–49 signed informed consents.

A 27-item questionnaire was developed and beta-tested on 10 sexually active women age 15–49, for comprehension and content validity. The questionnaire was designed to take 15 min to administer and covered the women’s demographics (age, education, occupation, marital status, number of children and religion) and her knowledge of contraceptive options and her personal contraceptive use.

Women eligible to participate in the study had to live in the KEHD for at least twelve consecutive months and have been educated on the MMC; speak English or pidgin; be willing to give informed consent; and to be at-risk of pregnancy, defined as not being pregnant at enrollment, being fertile and of reproductive age (15–49 years of age). In addition, they needed to have had sex in the past month or be planning on having sex in the next month with at least one fertile male partner and not consistently using any birth control. We defined inconsistent use of birth control as not having used contraception during every sexual encounter within 3 months before enrollment. The surveyors were trained on all aspects of the study, including exclusion criteria.

### Sample size determination

The sample size was calculated using the sample size formula for two proportions and confirmed with Epi info version 7. Based on the fact that we used cluster sampling in this study, a design effect of 2 was employed [11].

$$ \mathrm{N}=\mathrm{DEFF}\kern0.24em \mathrm{X}\kern0.24em 2\frac{\left| Zcrit\sqrt{2p\left(1-p\right)+ Zpwr\sqrt{P1\left(1-P1\right)+P2\left(1-P2\right)}}\right|2}{\mu^2}\kern1em \left[12\right] $$

Where p1 and p2 are pre-study estimates of the two proportions to be compared, D = |p1-p2| or minimum expected difference, = (+)/2 N. was the total sample size (sum of the sizes of both comparison groups), was the desired significance criterion and was the desired statistical power. The values used for the calculation included a statistical power of 0.80, = 0.842, Significance criterion of 0.05, = 1.960 and DEFF = 2 was the design effect [12]. The p1 = 0.2 % was the proportion of women using modern methods of contraception in the KEHD in 2011 [13]. p2 = 7.1 % was an assumption. We assumed that through home visits the contraceptive use would have increased by 6.9 % as was observed in Rwanda [14]. = 3.6, D =6.9. This gave a sample size N of 460.

Assuming a 20 % non-response rate, we therefore had a sample size of 552 (276 in each study group). After data collection, the response rate was 87.5 % therefore giving a sample size of 483 participants. From this sample size 262 women were recruited in the intervention health areas (IHA-home visits) and 221 participants were enrolled in the nonintervention health areas (NIHA-no-home visits).

### Sampling method

We used multistage cluster sampling method where a list of all the 20 health areas and their population of women of childbearing age was collected from the KEHD. The number of households per cluster was calculated from this population by dividing the total population by 6 (average size of a household in Cameroon was 6 persons per household) [15].

Next probability proportionate to size method was used to select the different clusters (communities) within the health areas. The representative sample of health areas within the district was done by calculating the sampling interval (the total number of households divided by the total number of clusters needed). A total of 25 clusters each was required for both the health areas implementing home visits and those with no home visits [13]. A random number between one and the sampling interval was chosen to represent our reference point. This random value was then added to the sampling interval to get the cluster positions. This was done continuously until we obtained the total number of clusters (communities) needed (25 clusters per group) (Table 1).

**Table 1:** Cluster Characteristics

Health areas with Home visit intervention					Health areas with no home visit intervention						
Health area	No. of women age 15–49 years	No. of households	cumulative	No. of clusters per health area	percentage particiants per cluster	community	No. of women age 15–49 years	No. of households	cumulative	# of cluster	percentage participants per cluster
Shisong	4396	733	733	4	16 %	Mbah	1690	282	282	1	4 %
Jakiri CMA	2725	454	1187	3	12 %	Sop	3184	531	813	4	16 %
Jakiri IHC	2913	486	1673	3	12 %	Ngorin	1035	173	985	2	8 %
Mbam	3306	551	2224	3	12 %	Mbonso	1697	283	1268	2	8 %
Mbokam	1323	221	2444	2	8 %	Verkovi	3307	551	1819	4	16 %
Ndzeng	1912	319	2763	2	8 %	Wvem	1016	169	1989	1	4 %
Nkar	1348	225	2988	1	4 %	Nkwaso	1408	235	2223	3	12 %
Lip	607	101	3089	1	4 %	Wainamah	1433	239	2462	3	12 %
Tartum	3420	570	3659	3	12 %	Mbiame	5066	844	3306	5	20 %
Ngendzen	411	69	3727	1	4 %						
Wasiber	1492	249	397	2	8 %						
Total	23853	3976			100 %			3306			100 %

Finally, a simple random sampling of the groups was selected by balloting among the clusters in order to identify the communities to be used for the study in each health area. Then an equal number of participants (first 11 women per cluster or community) was selected and one woman of childbearing age was interviewed per household. Also, we had a briefing meeting with health personnel already in the pilot project at the beginning of our study to ensure that those staff also understood clearly the objectives of our study.

We entered data into Microsoft Excel and analyzed with the statistical package STATA. The analysis focused on the impact of home visit involving family planning counseling, without receipt of contraception, and no home visits. In computing the socio-demographic characteristics of study participants, measures of central tendencies: mean and standard deviation were used while frequencies were used to compute level of education, religion, marital status, occupation and number of children. The Chi squared, student *t*-test and Anova were used to test for significant associations when appropriate. Binary logistic regression was used to measure the level of association and multiple logistic regressions were computed to control for confounders. Statistical significance was set at *p* < 0.05.

## Results

**Fig. 1:**
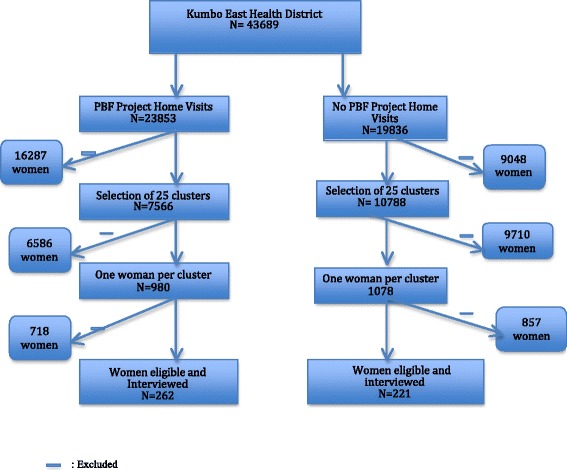
Consort diagram

Data were collected from February 1 to May 31, 2015. Selected homes were visited and the questionnaire was administered to eligible participants who consented to be part in the study. Data was collected on traditional holidays, market days and after 4 pm when women were at home after their daily activities. We could not rely on telephone numbers because majority of our patients did not have phones and the telephone network was usually difficult to go through During that period 552 participants were enrolled, but only 483 (87.5 %) were interviewed and completed the survey. 262 were studied in health areas with home visits and 221 women in health areas with no home visits (Fig. 1). The mean age of women in each of the groups was similar 30.40 (SD 8.57); median 30 years; range 15–48 years versus 30.49 (SD 7.84); median 30 years; range 16–49 years (*p* = 0.90) (Table 1). Over half (52 %) of study participants were Catholics (*p* < 0.03) and 44.7 % had secondary education or less (*p* < 0.06). There were no statistical differences in participant characteristics between the intervention and no intervention groups, including marital status (*p* < 0.25), employment status (*p* < 0.78) and having parity of 2 or more children (*p* = 0.54) (Table 2).

**Table 2:** Socio-demographic characteristics of study population

		Intervention (home visit)		
Variable		No (*N* = 221)	Yes (*N* = 262)	*P*-value
Age (years)				
	Age (±SD)	30.40 ± 8.57	30.49 ± 7.84	0.90
Education (N, %)				
	None	14 (6.33 %)	7 (2.67 %)	0.06
	Primary level	94 (42.53 %)	98 (37.40 %)	
	Secondary level	91 (41.18 %)	117 (44.66 %)	
	Tertiary level	22 (9.95 %)	40 (15.27 %)	
Religion (N, %)				
	Catholic	116 (52.49 %)	131 (50.00 %)	0.03
	Baptist	35 (15.84 %)	22 (8.40 %)	
	Presbyterian	32 (14.48 %)	53 (20.23 %)	
	Pentecostal	0 %	3 (1.15 %)	
	Muslim	38 (17.19 %)	53 (20.23 %)	
Marital status (N,%)				
	Single	39 (17.65 %)	48 (18.32 %)	0.25
	Married	164 (74.21 %)	195 (74.43 %)	
	separated/divorced	15 (6.79 %)	10 (3.82 %)	
	Widowed	3 (1.36 %)	9 (3.44 %)	
Occupation (N, %)				
	Unemployed	80 (36.20 %)	98 (37.40 %)	0.78
	Employed	141 (63.8 %)	164 (62.60 %)	
Number of children (N, %)				
	0–2	95 (42.99 %)	126 (57.01 %)	0.54
	3 > 5	120 (45.80 %)	142 (54.20 %)	

Nearly 6 out of 10, (59.92 %) of the participants in health areas with home visits reported current use of MMC compared to 46.61 % participants in the control group used MMC, (*p* = 0.003). The odds ratio was 0.7 (95 % CI, 1.19–2.46.). After adjusting for differences in regions, the aOR was 1.8 (95 % CI, 1.21–2.53).

In both areas, the most frequently used methods were behavioral methods, but their use was much less common in the intervention group (25.9 vs 38.1 %). The most common MMC used in the intervention area was injections (24.1 %) whereas, the male condom was the most commonly used MMC in the nonintervention area (25.8 %). In the intervention group, use of other MMC was significantly more frequent; pill use was higher than implant use (7.7 vs 1.8 %) (Table 3).

**Table 3:** Distribution of current use of MMC

intervention (home visit)								
Variable	no	yes	*p* value	OR	95 % CI	aOR	*p* value	95 % CI
Current use of MMC (N, %)								
No	118/221 (53.39 %)	105/262 (40.08 %)						
Yes	103/221 (46.61 %)	157/262 (59.92 %)	0.003	1.7 1	1.19–2.46	1.7 5	0.003	1.21–2.53
Current use of MMC by method (N, %)								
Natural	84/221 (38.01 %)	68/262 (25.95 %)						
Implant	4/221 (1.81 %)	12/262 (4.58 %)		3.71	1.14–12.01	3.56	0.029	1.08–11.73
IUD	7221 (3.17 %)	9/262 (3.44 %)		1.59	0.56–4.49	2.01	0.382	0.69–5.84
Tubal								
ligation	1/221 (0.45 %	2/262 (0.76 %)		2.47	0.22–27.83	2.82	0.464	0.23–34.77
Injectable	17/221 (7.69 %)	63/262 (24.05 %)		4.58	2.45–8.54	4.95	<0.001	2.63–9.34
Pills	17/221 (7.69 %)	26/262 (9.92 %)		1.89	0.95–3.77	2.04	0.071	1.01–9.34
male								
condoms	57/221 (25.79 %)	46/262 (17.56 %)		1.00	0.60–1.64	1.02	0.99	0.61–1.70
None	34/221 (15.38 %)	36262 (13.74 %)	<0.001	1.31	0.74–2.31	1.45	0.354	0.82–2.59
Frequency of use of MMC (N, %)								
Frequent	47/103 (45.63 %)	81/157 (51.59 %)						
Infrequent	56/103 (54.37 %)	76/157 (48.41 %)	0.35					

*aOR* adjusted odds ratio, *OR* odds ratio, *CI* Confidence Intervals

## Discussion

Our study has shown that MMC use was more common in areas served by PBF home visitors. The importance of PBF home visits to our study population stems from the fact that most women are farmers and are not readily at home to obtain information about MMC from local radio stations. This is consistent with other studies that have proved that use of social workers increase the use of MMC [16]. Mercer et al. in Bangladesh, did find that home visits increased the use of injectables from 5 to 10 % [17], as well as USAID report which found a significant increase in the use of injectables in programs that implemented home delivery of MMC [18]. The fundamental difference between our study and that in Bangladesh was that field workers in rural Bangladesh distributed injectables to women at home while in this study (KEHD) women had to go to health facilities to get the MMC.

However, our findings differ from a Rwandan study by Skile and colleagues; they found that home visit did not impact the use of MMC. It is possible that this may be due to the extreme equity gap seen between the two populations studied [14].

Melnick et al. in the USA did not find an association between the method of contraception used and home visits [19]. This difference could possibly be because our study recruited participants who were interested in delaying pregnancy using specific methods. In addition, we found that women in health areas with home visits were 3.6 times more likely to use implants compared to those without home visits. This was not consistent with the findings of Egede JO et al. in Nigeria who reported that the most commonly used forms of modern contraception were the barrier method (male condoms, 8.2 %, the oral contraceptive pill 3.0 %, injectables 2.5 %, and the intrauterine contraceptive device 2.0 % [20].

The use of MMC is responsible for improving maternal and infant health outcomes [7]. A global review of 172 studies by Ahmed and colleagues in 2012 calculated that the use of MMC prevented 272,040 maternal deaths, a 44 % reduction without which maternal deaths would have been 1.8 times higher than 2008 totals [21]. Cameroon has a maternal mortality rate of 680 women per 100,000 live births from pregnancy [6]. If all women with unmet needs of contraception were to use MMC, it is estimated that 373,000 unintended pregnancies could be prevented, and therefore, unplanned births, abortions and miscarriages could be reduced by ^3^/_4_; maternal deaths would be reduced by 1,300 and infant deaths by 13,000 annually [6]. This would substantiate many of the older millennium development goals.

Our study was based on a self-reported use of MMC, therefore they may be a risk of recall bias and reporting bias. An additional limitation is potential selection bias. Because we could not obtain personal information on participants until they consented to participate in the study, we have no information on those who refused to participate, nor do we have information on potential participants who were difficult to reach.

Other confounders may have impacted on our findings. Some of the strategies from the intervention pilot project may have spread into the control area. Some women that were interviewed in the areas not served by the pilot project said they had heard of MMC from home-based counselors. Similarly, HIV prevention campaigns conducted in all health areas promoted both male and female condoms and may also have increased the use of the modern method in the control area. However, these confounders would have decreased the impact of the pilot project. The fact that we observed a 70 % higher utilization of MMC in the PBF pilot project despite these confounders supports the effectiveness of this initiative. Extension of this project into other regions may well yield important benefits to the health of reproductive age women and their families. Future research that explores the involvement of religious leaders in these regions may identify ways to magnify these benefits.

## Conclusion

Utilization of MMC was significantly (1.7 times) higher in the area with PBF home visits than in areas without that service. While this indicates that the intervention has benefit, there may be other contributing factors.

## Abbreviations

IHA: Intenvention health areas
KEHD: Kumbo east health district
MMC: Modern methods of contraception
NIHA: Non intervention health areas
PBF: Performance based financing
STD: Sexually transmitted diseases
